# Immune Landscape and Classification in Lung Adenocarcinoma Based on a Novel Cell Cycle Checkpoints Related Signature for Predicting Prognosis and Therapeutic Response

**DOI:** 10.3389/fgene.2022.908104

**Published:** 2022-05-11

**Authors:** Jian Yang, Zhike Chen, Zetian Gong, Qifan Li, Hao Ding, Yuan Cui, Lijuan Tang, Shiqin Li, Li Wan, Yu Li, Sheng Ju, Cheng Ding, Jun Zhao

**Affiliations:** ^1^ Department of Thoracic Surgery, The First Affiliated Hospital of Soochow University, Suzhou, China; ^2^ Institute of Thoracic Surgery, The First Affiliated Hospital of Soochow University, Suzhou, China; ^3^ Department of Thoracic Surgery, The First Affiliated Hospital of Nanjing Medical University, Nanjing, China; ^4^ Department of Pathology, Affiliated Hospital of Nantong University, Nantong, China; ^5^ Department of Urinary Surgery, The First Affiliated Hospital of Soochow University, Suzhou, China; ^6^ Soochow University Laboratory of Cancer Molecular Genetics, Medical College of Soochow University, Suzhou, China

**Keywords:** lung adenocarcinoma, cell cycle checkpoints, prognostic signature, tumour immune microenvironment, therapeutic response

## Abstract

Lung adenocarcinoma (LUAD) is one of the most common malignancies with the highest mortality globally, and it has a poor prognosis. Cell cycle checkpoints play a central role in the entire system of monitoring cell cycle processes, by regulating the signalling pathway of the cell cycle. Cell cycle checkpoints related genes (CCCRGs) have potential utility in predicting survival, and response to immunotherapies and chemotherapies. To examine this, based on CCCRGs, we identified two lung adenocarcinoma subtypes, called cluster1 and cluster2, by consensus clustering. Enrichment analysis revealed significant discrepancies between the two subtypes in gene sets associated with cell cycle activation and tumor progression. In addition, based on Least Absolute Shrinkage and Selection Operator (LASSO) Cox regression, we have developed and validated a cell cycle checkpoints-related risk signature to predict prognosis, tumour immune microenvironment: (TIME), immunotherapy and chemotherapy responses for lung adenocarcinoma patients. Results from calibration plot, decision curve analysis (DCA), and time-dependent receiver operating characteristic curve (ROC) revealed that combining age, gender, pathological stages, and risk score in lung adenocarcinoma patients allowed for a more accurate and predictive nomogram. The area under curve for lung adenocarcinoma patients with 1-, 3-, 5-, and 10-year overall survival was: 0.74, 0.73, 0.75, and 0.81, respectively. Taken together, our proposed 4-CCCRG signature can serve as a clinically useful indicator to help predict patients outcomes, and could provide important guidance for immunotherapies and chemotherapies decision for lung adenocarcinoma patients.

## Introduction

Lung cancer is one of the leading threats to human health, with more than 1.8 million lung cancer cases globally, according to Global Cancer Statistics 2020 estimates (Sung et al., 2021). Non-small cell lung cancer (NSCLC) accounts for about 85% of lung cancers, and lung adenocarcinoma (LUAD) is the most common subtype of NSCLC, with a high degree of heterogeneity and aggressiveness (Thai et al., 2021). Despite improvements in multiple therapies, LUAD patients still have poor prognosis, due to local recurrence and distant metastasis (Tan and Tan, 2022). Therefore, it is necessary to develop a risk stratification method and find reliable molecular signature for early diagnosis, prognostic prediction, and treatment options of LUAD.

Tumours are a class of diseases in which cell cycle regulatory mechanisms are disrupted (Lugli et al., 2021). Cell cycle checkpoints serve as DNA surveillance mechanisms in the entire system of monitoring cell cycle processes, and play a central role in preventing the accumulation and reproduction of genetic mutations during cell division (Panagopoulos and Altmeyer, 2021). Importantly, some studies have indicated that most cell cycle control functions are essential for cancer cell survival (Walston et al., 2021; Liu et al., 2022). In cancer cells, DNA damage checkpoints are frequently damaged, allowing cells to continue dividing despite the accumulation of genetic errors (Huang and Zhou, 2020). Conversely, genes involved in DNA replication stress checkpoints in cancer cells rarely mutate, as many cancers increasingly rely on DNA replication stress checkpoints function to tolerate high levels of replication stress (Técher et al., 2017). Similarly, cancer cells rely on functional spindle assembly checkpoints to prevent catastrophic missegregation of chromosomes (Musacchio, 2015). Previous studies have shown that cell cycle checkpoints related genes have potential prognostic value in a variety of cancers, so targeting cell cycle checkpoints is therefore a promising strategy (Fei and Xu, 2018; Sonntag et al., 2021). It is feasible to establish a risk signature based on the cell cycle checkpoints to assess patients outcome and therapeutic efficacy. Notably, several risk signatures have been developed to explore the prognostic value of DNA damage repair and cell cycle progression-related genes (Chen et al., 2021a; Jiang et al., 2021). However, the predictive value of risk signature constructed using cell cycle checkpoints as a clinical indicator in lung adenocarcinoma is unclear.

Tumour cells survive and proliferate *in vivo via* evading recognition and attack by the body’s immune system through a variety of mechanisms (Jiang et al., 2019; Anichini et al., 2020). Recently, immunotherapies for lung adenocarcinoma, which stimulates specific immune responses to kill tumour cells, has become a hot topic (Bauml and Knepley, 2020). Bioinformatics analysis of tumour immune microenvironment, tumour mutation burden (TMB), and immune checkpoints expression levels can help predict immunotherapy efficacy and promote precision therapies.

In this study, we investigated the potential biological significance of cell cycle checkpoints in LUAD. Based on prognostic genes associated with cell cycle checkpoints, we identified lung adenocarcinoma patients in the TCGA database into two subtypes using consensus clustering. In addition, we constructed a risk signature using LASSO-Cox regression, to more accurately evaluate the clinical value of CCCRGs in LUAD. There were significant differences in patients outcomes, immune implication, chemotherapeutic efficacy, and gene mutation status between high- and low-risk groups. This study may shed new light on the molecular mechanisms underlying LUAD, and provides insights into personalized targeted therapies for LUAD patients. In the future, the technology may help doctors make better treatment decisions.

## Materials and Methods

### Data Collection and Processing

By cleansing and standardizing lung adenocarcinoma data from The TCGA dataset, we obtained gene expression profiles from 515 tumour samples and 59 cancer-adjacent normal tissues [log2 (TPM+1)]. Clinical information data was eventually collated and extracted from 500 tumor samples after deletion of the missing data and samples with 0 survival time. GSE31210, GSE10072, GSE27262, GSE68465, and GSE50081 were downloaded from the Gene Expression Omnibus (GEO) database (https://www.ncbi.nlm.nih.gov/geo/). Samples lacking survival data were deleted and all data were standardized and corrected for log2 (x+1). Mutation data were downloaded from the TCGA database (https://portal.gdc.cancer.gov/). Tumor mutation burden data were downloaded from the cBioPortal (https://www.cbioportal.org/). Differentially expressed genes (DEGs) were analysed using the R package “limma” for TCGA, GSE3120, GSE10072, GSE27262, and GSE68465 databases (Ritchie et al., 2015). (|FoldChange|>2, adjusted *p* < 0.05).

### Consensus Clustering and Molecular Subtypes of Cell Cycle Checkpoints Related Genes

Univariate Cox analysis of differentially expressed CCCRGs was performed to obtain 25 prognostic related CCCRGs (Ahmed et al., 2022). Unsupervised cluster analysis was performed using R package “ConsensusClusterPlus”, using agglomeration km clustered with a 1-Pearson correlating distribution, and resampling of 80% of the samples for 1000 repetitions (Wilkerson and Hayes, 2010). The optimal clustering was determined under cumulative distribution curve (CDF) and the rationality of clustering was further validated by principal component analysis (PCA).

### Enrichment Analysis

Differentially expressed genes between the two subtypes were obtained by R package “limma” (version 3.40.6) (Ritchie et al., 2015). Gene ontology (GO) and Kyoto Encyclopedia of Genes and Genome (KEGG) analyses were performed by R package “Cluster Profiler” (version 3.14.3) to obtain enrichment results (Cao et al., 2021). (*p* value <0.05, FDR <0.05) Gene Set Enrichment Analysis (GSEA) software (version 3.0) was obtained from the GSEA website. We downloaded c2.cp.kegg.v7.4.symbols.gmt (KEGG) and h.all.v7.4.symbols.gmt (Hallmark) gene sets from the Molecular Signatures Database (http://www.gsea-msigdb.org/gsea/msigdb/index.jsp). GSEA analysis was conducted in two subgroups, to assess relevant pathways and molecular mechanisms based on gene expression profiles and phenotypic subsets, with a minimum set of 5 genes, a maximum set of 5,000 genes, and 1,000 repetitions (*p* value <0.05, FDR <0.05) (Subramanian et al., 2005). Gene Set Variation Analysis (GSVA) calculated the enrichment score for each sample in the Hallmark gene sets using the R package “GSVA”, setting the minimum set at 5 and the maximum set at 5000 (Hänzelmann et al., 2013).

### Construction of Signature

In this study, we used the R package “glmnet” to integrate survival time, survival status, and gene expression data for regression analysis using the LASSO-Cox method. We also set up a 10-fold cross validation to get the best signature (Wu et al., 2021). Ultimately, we constructed a prognostic signature based on CCCRGs to predict survival in LUAD patients. The risk score was calculated as follows: risk score = (β1 × Gene1 Exp) + (β2 × Gene2 Exp) + . . . + (βi × Genei Exp) (Tibshirani, 1997; Friedman et al., 2010; Wang et al., 2019). Patients with LUAD were classified into high- and low-risk groups based on risk score. Kaplan-Meier analysis and ROC analysis were conducted to examine the applicability and stability of the model. In addition, we used R package “rms” to establish a nomogram using Cox regression to assess prognostic significance of clinicopathologic factors and risk score in LUAD samples (Iasonos et al., 2008).

### Tumour Immune Microenvironment and Immunotherapies Efficacy Prediction

We evaluated ESTIMATEScore, ImmuneScore, StromalScore, and TumorPurity using the ESTIMATE algorithm (Ma et al., 2021). The TIMER algorithm was used to evaluate tumour infiltration of 6 immune cell types (Li et al., 2017). Single-sample GSEA (ssGSEA) was applied to calculate immune infiltration of 24 immune cell types (Chong et al., 2021). In addition, we validated immune infiltration using the EPIC algorithm (Yang et al., 2021). Tumour mutation burden, calculated based on somatic non-synonymous mutations, is a potential biomarker of immunotherapies response (Chan et al., 2019). In addition, we extracted and analyzed the expression profile of immune checkpoints genes CTLA4, LAG3, TIGIT, PD1, PDL1, PDL2, and TIM3 in the TCGA database. Analysis of TMB and immune checkpoints genes can be used to evaluate the efficacy of immunotherapies.

### Prediction of Chemotherapeutic Efficacy

Using the R package “pRRophetic” and LUAD patients’ gene expression matrices, we predicted minimum drug inhibition concentrations (IC_50_) in both high- and low-risk groups, and ultimately obtained drugs with statistically significant IC_50_ values that could be potential candidates for LUAD treatments (Geeleher et al., 2014).

### Genetic Mutation Analysis

Somatic mutation analysis of LUAD samples was obtained from the TCGA database website in the “maf” format (Liu et al., 2018). Waterfall mapping was then performed using the “Maftools” package in the R software. Visualization results helped analyze and summarize the mutant genes (Mayakonda et al., 2018).

## Results

### Identification of Two Different Molecular Subtypes in Lung Adenocarcinoma Based on Cell Cycle Checkpoints Related Genes

**FIGURE 1:**
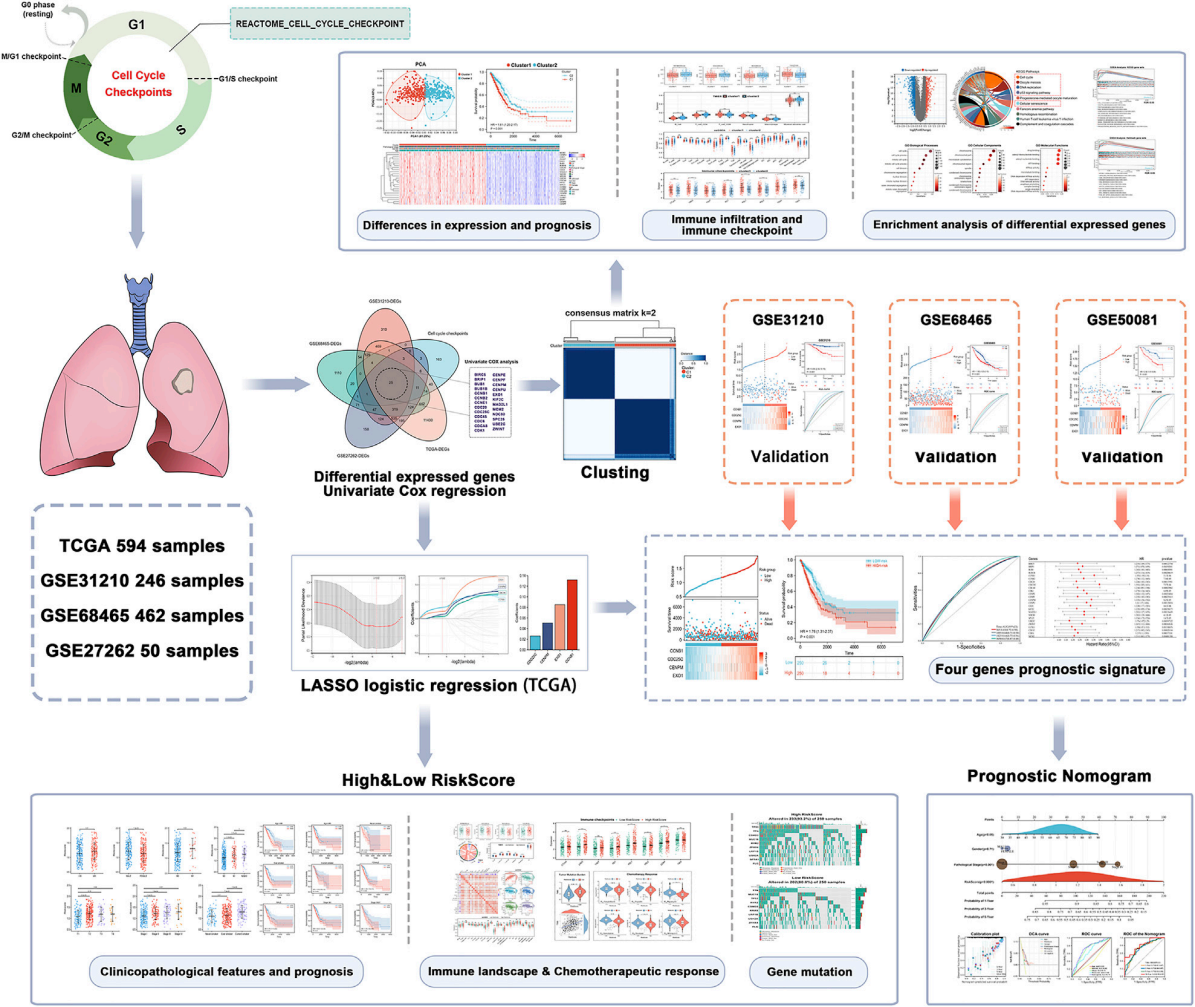
Flow chart of the data analyzing process.

**FIGURE 2:**
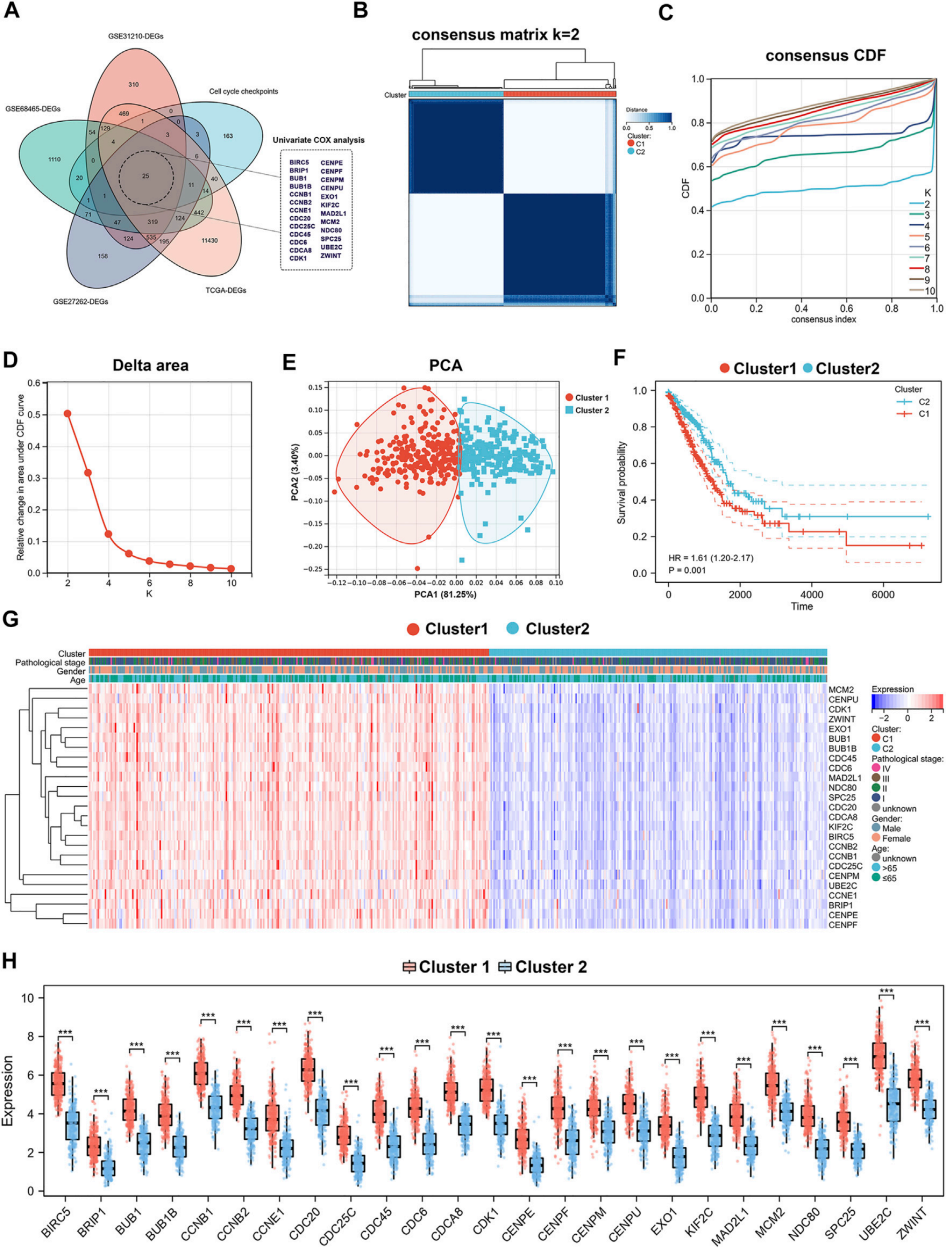
Identification of cell cycle checkpoints-associated subtypes by consensus clustering. **(A)** Prognostic associated differential genes obtained through Venn diagram. **(B–D)** Consensus clustering analysis for 25 genes in 515 LUAD samples (k = 2). **(E)** PCA of cluster1 and cluster2. **(F)** Kaplan-Meier curve of OS in the two subgroups. **(G**,**H)** Heatmap and histogram visualizing the expression of 25 CCCRGs in two subtypes.

The flow diagram of our present study is illustrated in Figure 1. Differentially expressed genes were obtained from four databases: TCGA-LUAD, GSE31210, GSE72094, and GSE27262. Cell cycle checkpoints related genes were derived from the Reactome_Cell_Cycle_Checkpoint gene set. Overlapping genes were identified by intersection of these differentially expressed genes with cell cycle checkpoints related genes. Then, 25 prognostic relevant CCCRGs were identified using univariate Cox regression analysis (Figure 2A). Based on consensus clustering of these genes, we subdivided LUAD patients in the TCGA cohort into subgroups (Figure 2B). When the cluster number K = 2, the clustering stability was the best (Figures 2C,D). In two subtypes, 280 patients were classified as cluster1, and 235 patients were classified as cluster2. We further validated the sample classification of cluster1 and cluster2 using principal component analysis (Figure 2E). By comparing the prognostic differences between cluster1 and cluster2, we found that patients in cluster1 had significantly worse prognosis (Figure 2F). In addition, expression of 25 CCCRGs were obviously different in both subtypes, and were generally higher in cluster1 (Figures 2G,H). we found LUAD patients in cluster1 had higher pathological stages than in cluster2 *via* volcano map. Furthermore, we compared the age and gender differences between the two subtypes, and found that 46.44% of patients were older than 65 in cluster1, while 58.08% of patients were older than 65 in cluster2. In terms of gender, 47.86% of patients in cluster1 were female, while 60.43% of patients in cluster2 were female (Supplementary Figures S1A,B). When we investigated the prognostic difference between the two subtypes in terms of age and gender, we found that cluster1 had a worse prognosis in patients older than 65, while no significant difference between cluster1 and cluster2 in patients younger than 65. In addition, in both sexes, cluster1 had a worse prognosis than cluster2 (Supplementary Figure S1C).

### Two Subtypes of Lung Adenocarcinoma Patients Exhibited Different Immune Landscapes

**FIGURE 3:**
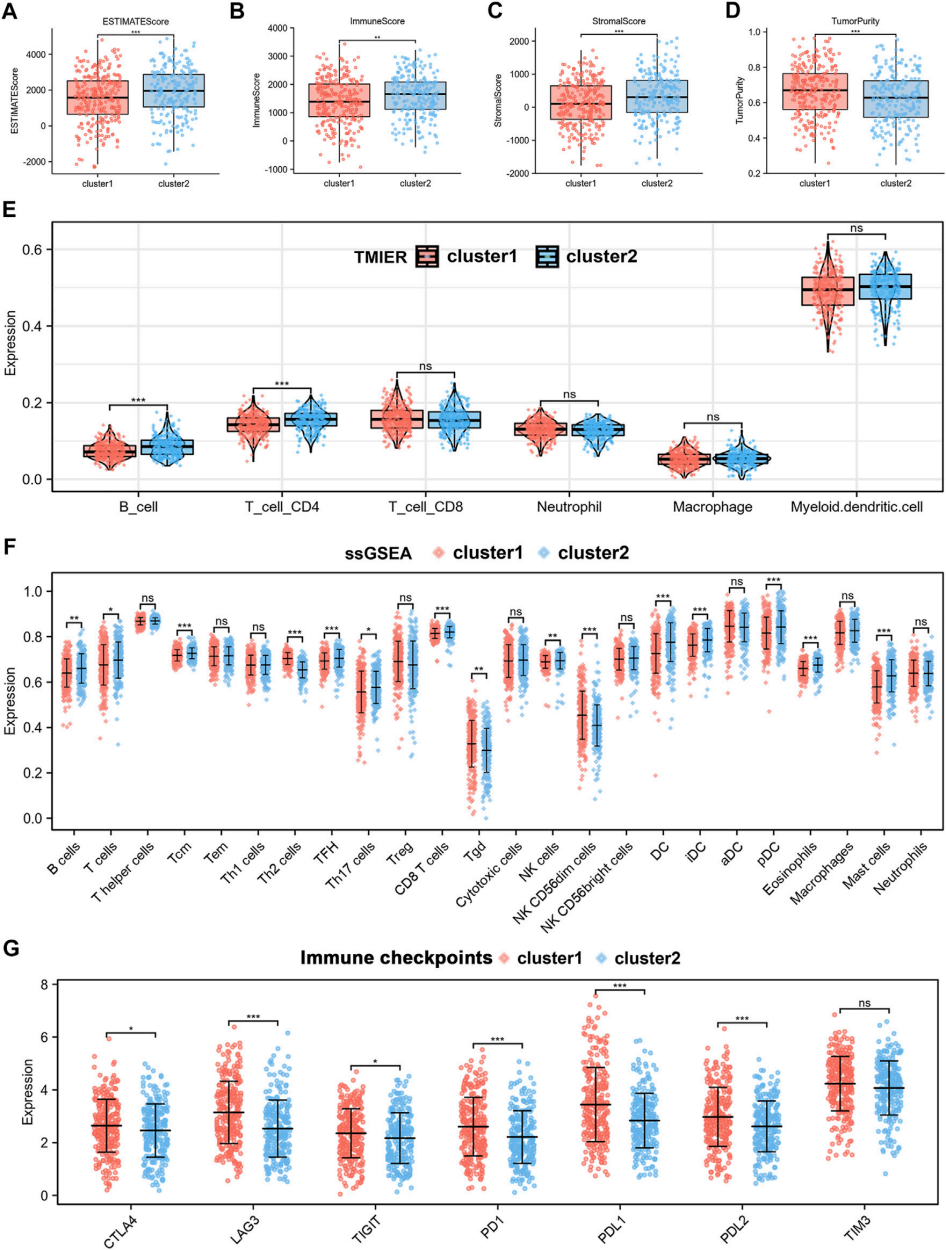
Analysis of the TIME and the expression of immune checkpoints in two subtypes. **(A**–**D)** ESTIMATE score, ImmuneScore, StromalScore and TumorPurity calculated by ESTIMATE algorithm. **(E)** Immune infiltration of 6 immune cell types using TIMER algorithm. **(F)** Immune infiltration of 24 immune cell types using ssGSEA algorithm. **(G)** Differences in expression of immune checkpoints between the two subtypes.

We performed immunoinfiltration analyses of the two different subtypes, and the results from the ESTIMATE algorithm showed that cluster1 had lower ESTIMATEScore, ImmuneScore, StromalScore, while higher TumourPurity compared to cluster2 (Figures 3A–D). The results of the TIMER algorithm showed that cluster1 has a lower abundance of B Cells and CD4+T Cells (Figure 3E). The results of the EPIC algorithm showed that cluster1 has a lower abundance of B Cells, CD4+T Cells, endothelial, and NK cells (Supplementary Figure S3A). In addition, immune infiltrating landscapes derived using ssGSEA algorithms were significantly different between cluster1 and cluster2. As shown in Figure 3F, B cells, T cells, Tcm cells, TFH cells, Th17 cells, CD8^+^ T cells, NK cells, DC cells, iDC cells, pDC cells, and Mast cells had lower immune status in cluster1, while Th2 cells, Tgd, and NK CD56dim cells had higher immune status in cluster1. Furthermore, we compared immune checkpoints expression across the two subtypes and found that CTLA4, LAG3, TIGIT, PD-1, PD-L1, PD-L2 were significantly higher in cluster1 than in cluster2, as shown in Figure 3G. Taken together, these results suggested that the cluster1 may be more favourable to tumour immune escape.

### Identification of Differentially Expressed Genes and Functional Enrichment Analysis in Two Subtypes

**FIGURE 4:**
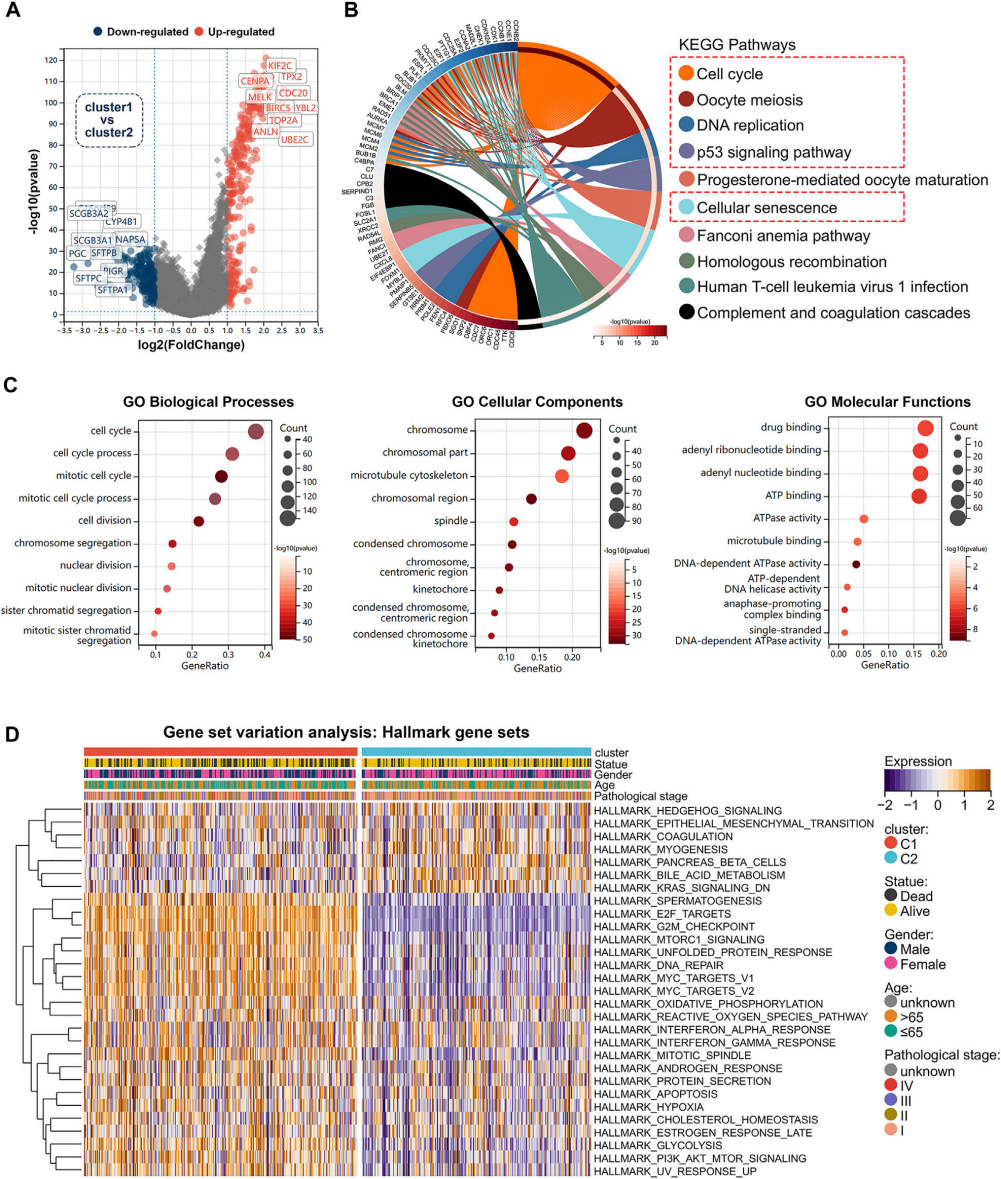
Functional enrichment analysis between two subtypes. **(A)** Volcano map showed differentially expressed genes between two subtypes in TCGA cohort. **(B)** Differentially expressed genes were selected for KEGG analysis (|FC|>2, *p* < 0.05). **(C)** GO analysis of differentially expressed genes (|FC|>2, *p* < 0.05). **(D)** Heat map showed difference in GSVA scores between two subtypes.

We analyzed differentially expressed genes between cluster1 and cluster2 using the R package “limma” (version 3.40.6). The volcano plot showed up-regulated and down-regulated genes (|FoldChange| >2) (Figure 4A).The potential biological mechanism was investigated by functional enrichment analysis. We selected 451 differentially expressed genes for KEGG and GO analysis (|FoldChange| >2) (Supplementary Table S1; Supplementary Table S2). KEGG results revealed that differentially expressed genes were enriched in cell cycle-related pathways (Figure 4B), such as cell cycle, oocyte meiosis, DNA replication, p53 signalling pathway, and cellular senescence. Furthermore, we found that the differentially expressed genes between the two subtypes were enriched for a variety of biological processes. Figure 4C illustrated the results of GO enrichment analysis, which can be classified into biological processes, cellular components, and molecular functions. GSEA analysis was carried out using the KEGG and Hallmark gene sets (Supplementary Table S3; Supplementary Table S4), which revealed that activation of cell cycle was significantly enhanced in cluster1 compared to cluster2, including cell cycle, oocyte meiosis, P53 signalling, MTORC1 signalling, G2M checkpoint, MYC targets, Glycolysis, DNA repair, E2F targets, PI3K-AKT-MTOR signalling, and other associated pathways (Supplementary Figures S2A,B). To further explore the differences in biological pathways between two different cell cycle checkpoints related subtypes, we used the GSVA algorithm to calculate Hallmark gene sets enrichment scores (Supplementary Figure S2C; Supplementary Table S5). Notably, by comparing the differences in GSVA scores between the two subtypes, we obtained 29 statistically significant biological pathways (Figure 4D). These results suggested that cluster1 is significantly associated with tumor initiation and progression, and may lead to poor prognosis in lung adenocarcinoma patients by affecting cell cycle and proliferation-associated signalling pathways.

### A Risk Signature Was Built by Cell Cycle Checkpoints Related Genes in TCGA Database

**FIGURE 5:**
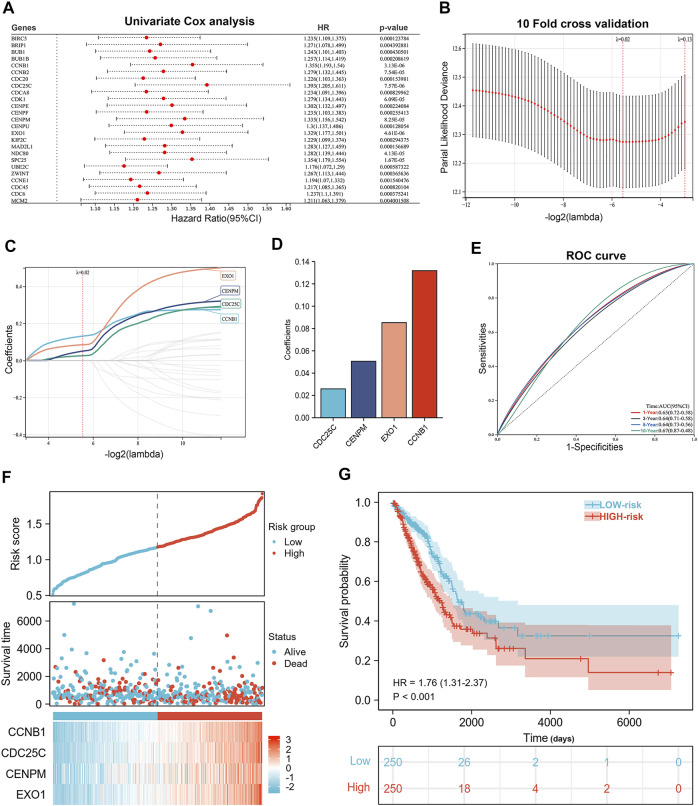
Construction of the prognostic signature. **(A)** Forestplot showed 25 prognostic associated CCCRGs obtained by univariate Cox regression analysis. **(B–D)** LASSO regression analysis and 10-fold cross-validation were performed to calculate the best lambda and identify the four most significant prognostic genes. **(E)** ROC results from a 4-genes prognostic model were used to analyze patients’ 1-, 3-, and 5-year overall survival. **(F)** Risk score, outcome status, gene expression profiles were shown in the training cohort. **(G)** Kaplan-Meier curve for OS in training cohort based on risk score.

**FIGURE 6:**
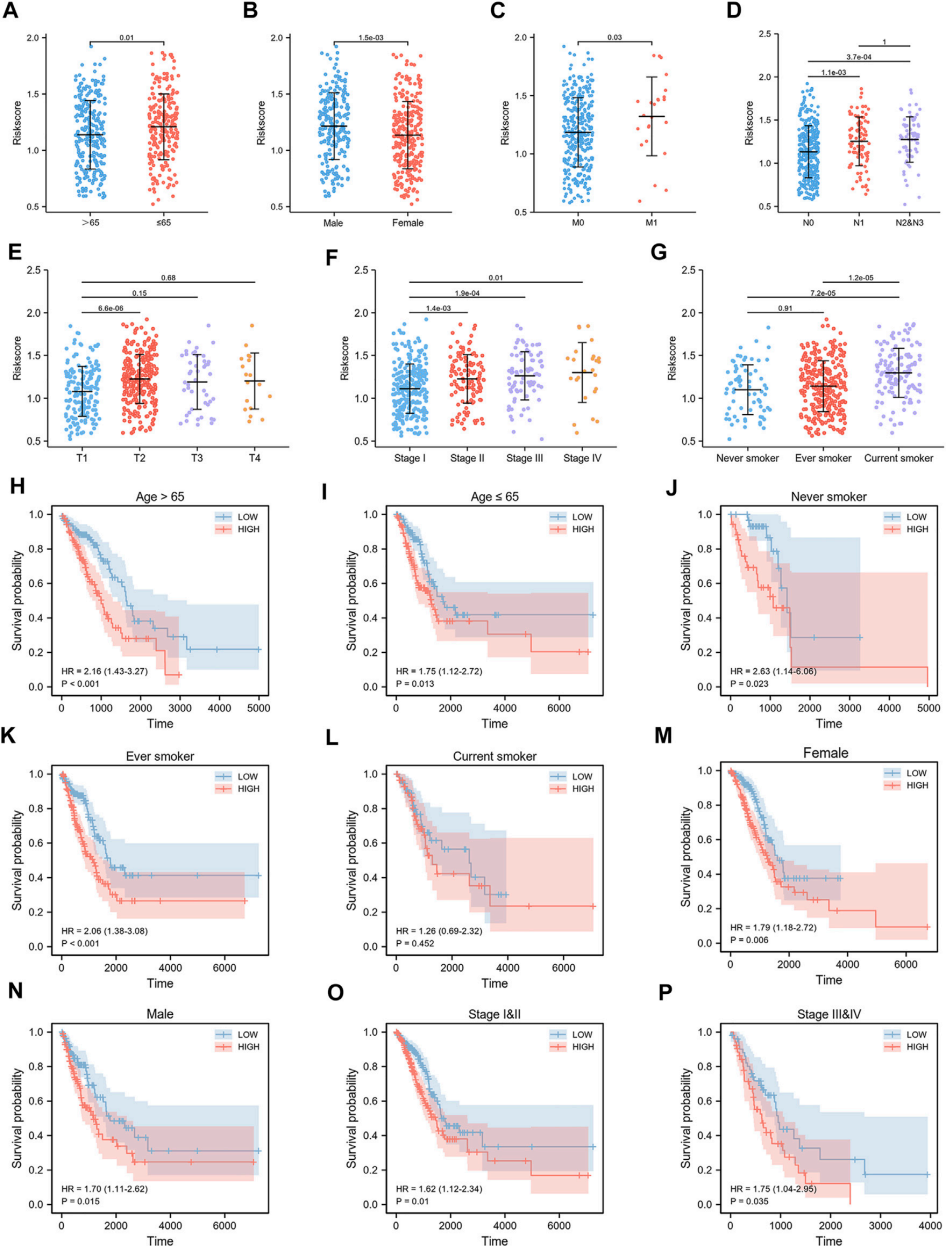
Relationship between risk score and clinicopathological features. Risk score differed by age **(A)**, gender **(B)**, smoking status **(G)**, TNM stage **(C–E)**, and pathological stage **(F)**. Survival curves of LUAD patients by age **(H**,**I)**, smoking status **(J**–**L)**, gender **(M,N)** and pathological stage **(O**,**P)** were obtained by comparing high-risk group with low-risk group.

We constructed a risk signature to predict the prognostic value of CCCRGs in lung adenocarcinoma. Figure 5A showed the results of the univariate Cox analysis. Then, we performed a LASSO regression analysis using 25 overall survival-related (OS) CCCRGs (Figure 5B). A risk signature with four genes was selected, using optimal lambda values (CCNB1, CDC25C, CENPM, EXO1) (Figures 5C,D). In addition, according to the coefficients of these four genes, we calculated the risk score of each LUAD patient as follows: risk score = (0.131810530210757 × CCNB1 Exp) + (0.0258950480925646 × CDC25C Exp) + (0.0505775207458941 × CENPM Exp) + (0.0852753768507349 × EXO1 Exp). Regarding the diagnostic efficiency of the risk signature, ROC curves presented acceptable assessment results (Figure 5E). Based on risk score, our prognostic model successfully classified LUAD patients into high- and low-risk groups. Figure 5F showed differences in the expression and survival status of four candidate genes between high- and low-risk groups. By mapping the Kaplan-Meier survival curve, we were able to conclude that the OS of 250 patients in the high-risk group was worse than that of 250 patients in the low-risk group (*p* < 0.001) (Figure 5G). We then compared differences in clinicopathologic factors between high and low risk groups. Results revealed that patients with age ≤65 had higher risk score (Figure 6A), male had higher risk score (Figure 6B), current smokers had higher risk score than never smokers (Figure 6G), and those with higher TNM stages and pathological stages tended to have higher risk score (Figures 6C–F). In addition, prognostic analysis of various clinicopathological factors revealed poor outcomes in high-risk group (Figures 6H–P).

### Validation of Four Genes Prognostic Signature in Gene Expression Omnibus Cohort

**FIGURE 7:**
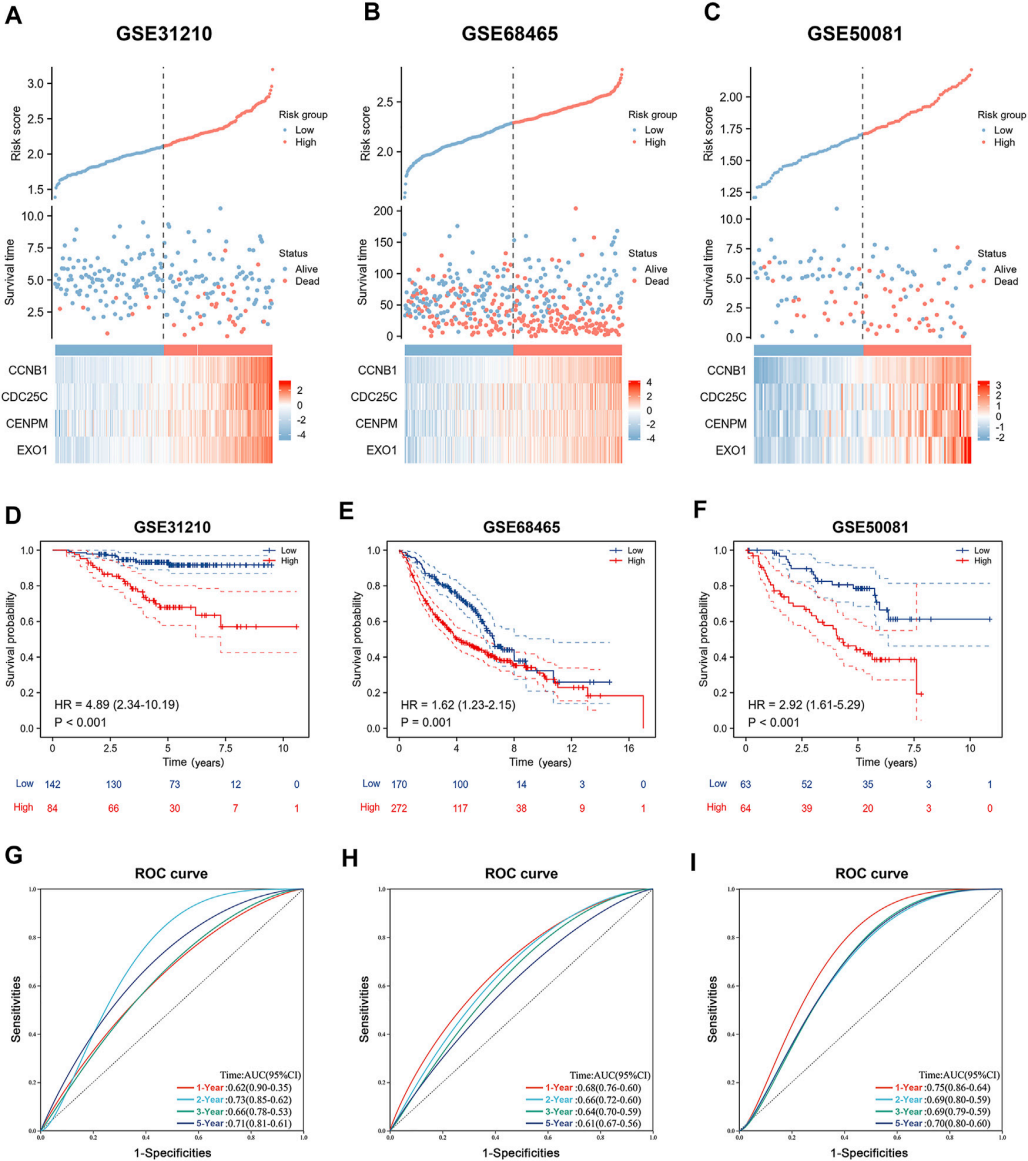
Validation of the prognostic signature. **(A–C)** Risk score, outcome status, gene expression profiles were shown in the GSE31210 cohort, GSE68465 cohort and GSE50081 cohort. **(D–F)** Kaplan-Meier curve for OS in validation cohort based on risk score. **(G–I)** Time-independent ROC curves of the risk score for predicting the 1-, 3-, and 5-year overall survival in the validation cohort.

To better verify the predictive ability of the prognostic signature we constructed, we calculated the risk score of LUAD patients in the GSE31210, GSE68465, and GSE50081 databases, using the same formula: risk score = (0.131810530210757 × CCNB1 Exp) + (0.0258950480925646 × CDC25C Exp) + (0.05057720458941 × CENPM Exp) + (0.0852753768507349 × EXO1 Exp). Based on the risk score, patients were divided into high-risk and low-risk groups. Heat maps showed that four genes in GSE31210, GSE68465, and GSE50081 had higher expression in high-risk groups (Figures 7A–C). The Kaplan-Meier survival curves revealed worse outcomes in high-risk groups across all three GEO databases (Figures 7D–F). As shown in Figures 7G–I, the AUC values of time-dependent ROC curves at 1-, 3-, and 5-years show acceptable assessment results. In addition, we performed a comprehensive bioinformatics analysis of all four genes involved in the development of prognostic signature, to further understand their expression in cancers. By analysing the TCGA database, we found that CCNB1, CDC25C, CENPM, and EXO1 were highly expressed in almost all of the 33 cancers (Supplementary Figures S4A–D), and we also noticed significant high expression in LUAD (Supplementary Figure S5C). By extracting immunohistochemistry data from the Human Protein Atlas (HPA) database, we found that CCNB1 and CENPM are highly expressed (at protein level) in LUAD and associated with poor prognosis (Supplementary Figures S5A,B). Finally, a comprehensive analysis of TCGA, GEPIA and Kaplan-Meier Plotter databases revealed that high mRNA expression of CCNB1, CDC25C, CENPM, and EXO1 was associated with poor survival in lung adenocarcinoma patients (Supplementary Figures S6A–D).

### Risk Signature Was Associated With Tumor Immune Microenvironment in Lung Adenocarcinoma

**FIGURE 8:**
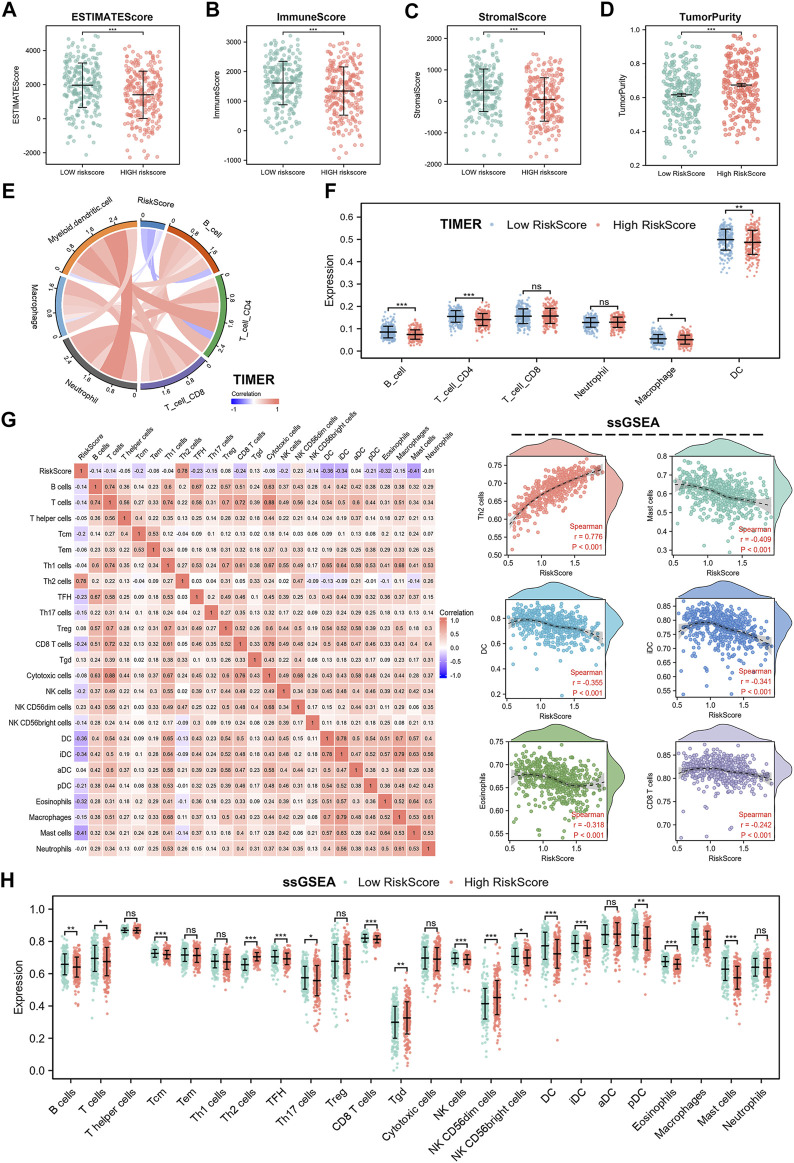
Immune landscape in cell cycle checkpoints related signature. **(A–D)** ESTIMATE score, ImmuneScore, StromalScore and TumorPurity calculated by ESTIMATE algorithm. **(E,F)** Immune infiltration of 6 immune cell types using TIMER algorithm. **(G)** Relationship between risk score and abundance of 24 immune cell types. **(H)** Differences in abundance of 24 immune cells between high- and low-risk group.

Spearman analysis was used to analyze the relationship between risk score and immune cell subpopulations. First, we employed the ESTIMATE algorithm to assess differences between high- and low-risk scores. The results showed that the high-risk group had lower ESTIMATEScore, ImmuneScore, and StromalScore, while higher TumorPurity compared to the low-risk group (Figures 8A–D). Subsequently, we used TIMER algorithm to analyze the abundance of six immune cell types in the high- and low-risk groups. Results showed that B cells, CD4+T cells, macrophages, and myeloid DC cells were less abundant in the high-risk group, which suggested that cell cycle checkpoints-related genes may promote tumor progression by suppressing anti-tumor immune system activation (Figure 8F). Furthermore, Figure 8E also showed negative correlation between risk score and immune cells infiltration. To further investigate the impact of risk score on the tumor immune microenvironment, we used the EPIC and ssGSEA algorithms. EPIC algorithm displayed that the high-risk group had lower abundance of B cells, CD4+T cells, endothelial cells, and NK cells than low-risk group (Supplementary Figure S3B). In addition, risk score was negatively associated with immune cells infiltration (Supplementary Figures S3C–E). The ssGSEA results showed a negative correlation between risk score and most of the 24 immune cell types, such as CD8+T cells, DC cells, iDC cells, eosinophils, Mast cells, and B cells (Figure 8G), while positively correlated with few immune cell types, such as Th2 cells. Notably, we also compared abundance differences between high- and low-risk groups across 24 immune cell types. We found higher abundance of B cells, T cells, Tcm cells, TFH cells, Th17 cells, CD8+T cells, NK cells, NK CD56bright cells, DC cells, iDC cells, pDC cells, eosinophils, macrophages, and mast cells in high-risk group, while lower abundance in Th2 cells, Tgd cells, and NK CD56dim cells in low-risk group (Figure 8H). However, there was no statistical difference between the high- and low-risk groups for T helper cells, Tem cells, Th1 cells, Treg cells, cytotoxic cells, aDC cells, and neutrophils. All of these results confirmed that LUAD patients in high-risk group exhibit a propensity for immune escape, indicating poor prognosis.

### Differences in Immunotherapies and Chemotherapies Responses, and Gene Mutations in Lung Adenocarcinoma Patients With High or Low Risk Score

**FIGURE 9:**
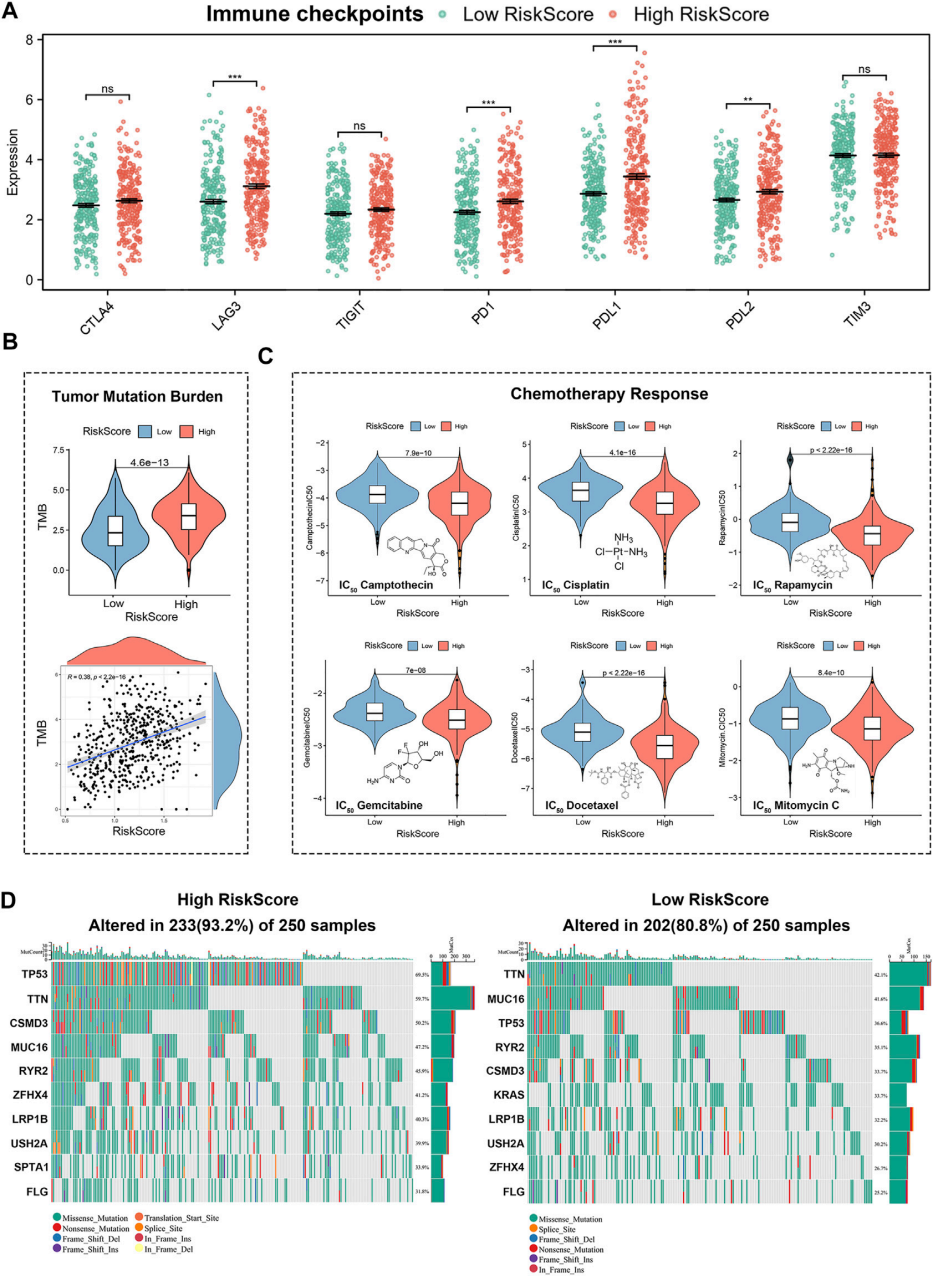
Gene mutation and response prediction for immunotherapies and chemotherapies in 4-genes risk signature. **(A)** Differences in expression of immune checkpoints between high-risk group and low-risk group. **(B)** Relationship between risk score and tumor mutation burden (TMB). **(C)** Differences in chemotherapies between high and low risk groups. **(D)** Status of mutations between high- and low-risk groups.

Immune checkpoints’ expression were strongly associated with immunotherapy efficacy. By comparing immune checkpoints expression between high- and low-risk groups, we found higher expression of LAG3, PD-1, PD-L1, and PD-L2 in the high-risk group, suggesting that LUAD patients with high risk scores may achieve better immunotherapy outcomes (Figure 9A). To further analyze the relationship between risk score and immunotherapies, we calculated the tumour mutation burden. Tumor mutation burden as a novel marker for evaluating the efficacy of PD-1 antibody therapy has been demonstrated in the treatment of cancers with mismatch repair defects. As shown in Figure 9B, LUAD patients in the high-risk group had higher tumour mutation burden, which meant patients with high risk scores are more susceptible to PD-1 antibody therapy. In addition, we performed IC_50_ analysis in high- and low-risk groups to screen for effective chemotherapies. The estimated IC_50_ of Camptothecin, Cisplatin, Rapamycin, Gemcitabine, Docetaxel, and Mitomycin C were significantly higher in the low-risk group than in the high-risk group. It suggested that lung adenocarcinoma patients in the low-risk group are more resistant to chemotherapies (Figure 9C). Finally, we analyzed differences in gene mutations between high- and low-risk groups (Figure 9D). As shown by the results, the most common types of mutations in the high-risk group in descending order were TP53, TTN, CSMD3, MUC16, RYR2, ZFHX4, LRPIB, USH2A, SPTA1, and FLG, while the most common types of mutations in the low-risk group in descending order were TTN, MUC16, TP53, RYR2, CSMD3, KRAS, LRP1B, USH2A, ZFHX4, and FLG.

### Construction and Validation of a Predictive Nomogram

**FIGURE 10:**
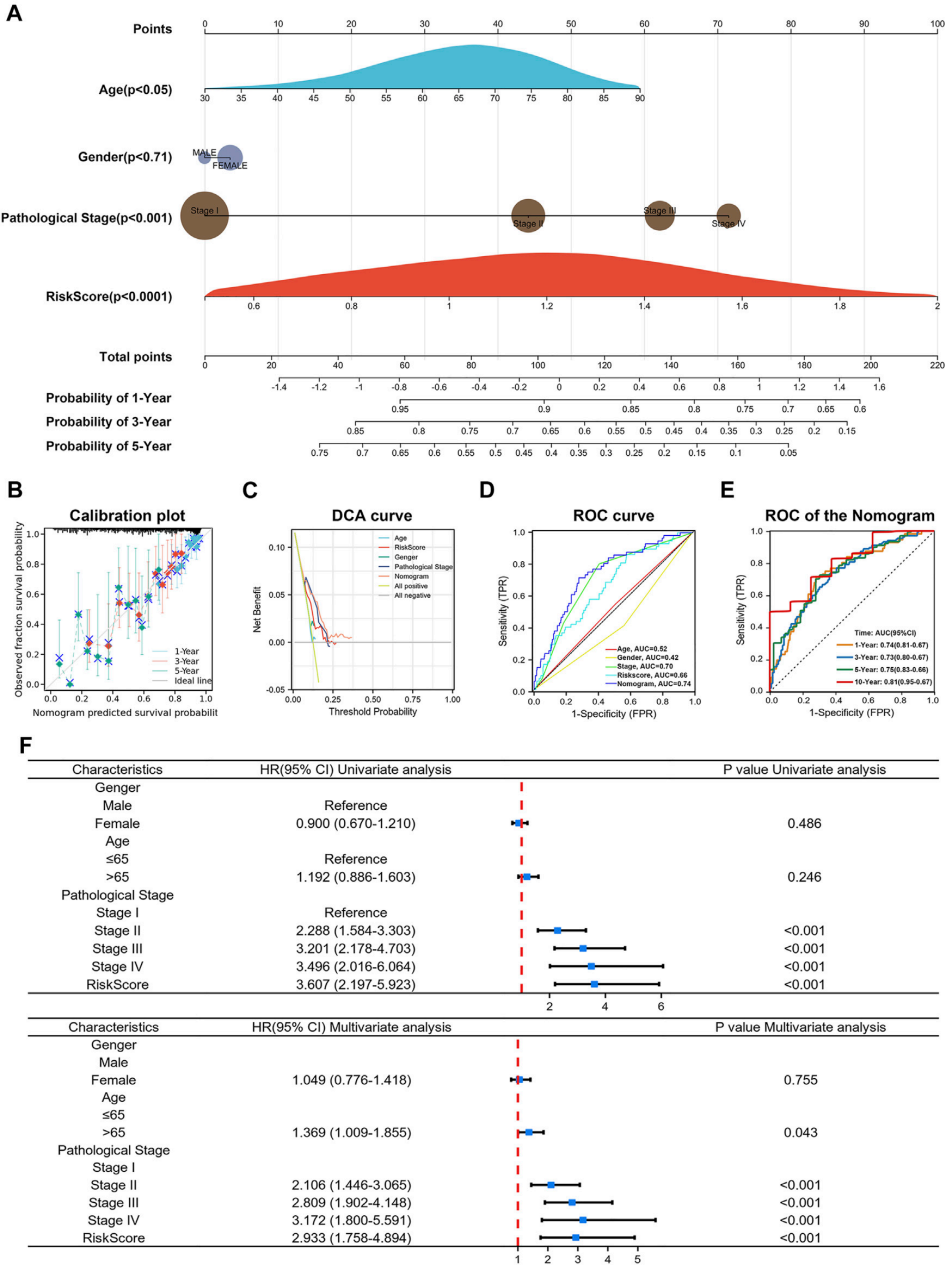
Construction of nomogram. **(A)** Nomogram showed risk score’s efficacy in predicting 1-, 3-, and 5-year OS after combining patients age, gender, and pathological stage. **(B–E)** Calibration plot, DCA curve and ROC curve showed acceptable accuracy. **(F)** Univariate Cox analysis and multivariate Cox analysis of risk score.

In addition, to further validate the usability and clinical applicability of the prognostic signature, we developed a predictive nomogram for 1-, 3-, and 5-year OS using the TCGA database. The nomogram integrated clinicopathologic factors such as age, gender, pathological stages with the risk score (Figure 10A **)**. (C-index = 0.70, *p* < 0.0001) The calibration curve of the nomogram showed good agreement between the predicted survival and the observed survival (Figure 10B). Decision curve analysis (DCA), and ROC curve also had acceptable accuracy (Figures 10C,D). The AUC value of the nomogram in the 1-, 3-, 5-, and 10-year overall survival were 0.74, 0.73, 0.75, and 0.81, respectively, which demonstrated excellent predictive efficacy (Figure 10E). Results from univariate Cox analysis and multivariate Cox analysis in LUAD patients revealed that the risk score was an independent prognostic factor (Figure 10F). Taken together, risk score, combined with other clinical parameters, can improve the model’s predictive accuracy. These results suggested that a risk signature based on CCCRGs can reliably and accurately predict outcomes in LUAD patients.

## Discussion

Cell cycle regulation in cancer cells is aberrant, with cancer cells receiving signals of continuous proliferation that drive continued cell division. A growing body of research has shown that this persistent cell division is driven not by uncontrolled cell cycle progression, but by mutations in signalling pathways that block apoptosis and initiate cell cycle exit (Chen et al., 2009; Matthews et al., 2022). Cell cycle checkpoints act as monitors of cell cycle activity, ensuring the integrity of the number of chromosomes and the proper functioning of the cell cycle (Matthews et al., 2022). Furthermore, aberrant expression of checkpoints genes in the cell cycle has been investigated and validated as an important factor involved in the pathogenesis and progression of LUAD (Keijzers et al., 2018; Zhang et al., 2018; Xiao et al., 2022).

Cell cycle checkpoints include DNA damage checkpoints, DNA replication stress checkpoints, and spindle assembly checkpoints. The primary role of DNA damage checkpoints in response to DNA damage is to prevent the accumulation and reproduction of genetic errors during cell division (Bednarski and Sleckman, 2019). DNA replication stress checkpoints only work in stage S, and their important function is to prevent DNA damage caused by replication stress (Li et al., 2022). DNA replication stress checkpoints control cell cycle progression by limiting CDK activity. The spindle assembly checkpoints (SAC) function at the M stage to ensure that the replicated DNA is equally distributed between the two daughter cells (Manic et al., 2017). Because the cell cycle is an extremely delicate regulatory process, endless division also poses fundamental challenges for cancer cells, which also need a number of cell cycle checkpoints to maintain their proliferative function. It is therefore realistic to explore the potential role of cell cycle checkpoints in LUAD and their impact on patient survival and treatment. Furthermore, due to the extremely complex tumor immune microenvironment of lung adenocarcinoma, the role of cell cycle checkpoints in regulating the tumor immune microenvironment requires further investigation (Egloff et al., 2006; Chen et al., 2020).

Previous studies have explored the potential value of huge cell cycle related genes in predicting survival in cancer patients. Yongfeng Hui et al. investigated the prognostic value of cell cycle progression-derived genes in hepatocellular carcinoma (HCC) (Hui et al., 2021). Wai Hoong Chang et al. explored the prognostic value of DNA repair genes in pan-cancer and confirmed that DNA repair genes were associated with dysregulation of cell cycle and hypoxia (Chang and Lai, 2019). Fangyu Chen et al. integratedly investigated the predictive value of cell cycle-related and immune-related genes in lung adenocarcinoma (Chen et al., 2021b). HCC can be divided into two subtypes with different molecular and clinical characteristics based on DNA damage repair related genes (Lin et al., 2021). Zhiyuan Zhang et al. constructed a robust signature based on the cell cycle-related genes in colon cancer (Zhang et al., 2021). However, most prognostic models were constructed using broad cell cycle related genes. Cell cycle checkpoints, a specific set of genes, had not been investigated for lung adenocarcinoma classification or survival prediction. Therefore, this study specifically targeted cell cycle checkpoints, a gene set that plays an important role in tumor activation and tumor immune microenvironment regulation (Chen et al., 2020). Further study of cell cycle checkpoints will facilitate targeted therapies and provide valuable recommendations for immunotherapy and chemotherapy options.

In this study, we performed a univariate Cox regression analysis of differentially expressed cell cycle checkpoints related genes in lung adenocarcinoma and finally identified 25 prognostic cell cycle checkpoints-related genes. Based on the expression of these genes, lung adenocarcinoma patients were categorized into two molecular subtypes using an unsupervised consensus clustering approach. The two subtypes had different prognostic states and immune phenotypes. In addition, different immunophenotypes can help guide specific immunotherapies. Of the two subtypes we identified, cluster1 had a worse prognosis and a reduced abundance of immune infiltration that promoted tumor escape. Cluster2, on the other hand, corresponded to a higher level of immune infiltration and was characterized by anti-tumor immunity. Notably, cluster1 expressed high levels of immunohibitors, suggesting a potential for better efficacy of immunotherapies. Upon enrichment analysis of both subtypes, we found that cluster1 significantly enriched for proliferation-associated signalling pathways such as E2F targets (Kent and Leone, 2019), G2M checkpoint (Smith et al., 2020), MTORC1 signalling (Carroll, 2020), MYC targets (Dang, 2012), PI3K-AKT-MTOR signaling (Polivka and Janku, 2014). We also noted that cluster1 has a higher GSVA enrichment score for the interferon response pathways than cluster2. Interferon signalling pathways were balanced between immune cells and tumor cells. Manipulating interferon signals could lead to more effective cancer immunotherapies (Benci et al., 2019). Furthermore, we found that cluster1 has higher glycolysis, hypoxia, and reactive oxygen species enrichment levels. Metabolic stress originating from mitochondria can accelerate cell differentiation in the absence of oxygen. Increased levels of reactive oxygen species (ROS) in T cells resulted in severe T cell dysfunction or exhaustion (Scharping et al., 2021). Therefore, reducing T cell ROS levels and alleviating tumor hypoxia can effectively block T cell functional immune exhaustion and achieve synergistic anticancer effects of tumor immunotherapies. Significantly, cluster2 was dramatically associated with downregulation of the KRAS signalling pathway. These results suggested that cluster2 may have lower mutation levels and better prognosis than cluster1.

To further investigate the prognostic effects of cell cycle checkpoints related genes on survival and treatment response, we performed LASSO-Cox regression analysis on 25 prognostic cell cycle checkpoints-related genes. Four genes most associated with prognosis were obtained: CDC25C, CENPM, EXO1, and CCNB1. Based on these four genes, we constructed a prognostic signature in the TCGA database. Patients with high risk scores died more often and had significantly worse outcomes than patients with low risk scores. To validate the reliability of the established signature, we validated the efficacy of the prognostic signature using three external validation sets (GSE31210, GSE68465, GSE50081), and obtained results consistent with the training set. Time-dependent ROC curves also showed good predictive accuracy.

Since cell cycle checkpoints were remarkably related to the tumor immune microenvironment, we also explored the association of risk scores with the tumor microenvironment. By using ESTIMATE, TIMER, EPIC, and ssGSEA algorithms, we found that high risk scores corresponded to low immune infiltration abundance, and low risk scores corresponded to high immune infiltration abundance. These results suggested that lung adenocarcinoma patients with high risk scores were more susceptible to tumor immune escape (Zeng et al., 2020). Therapies targeting immune checkpoints have developed significantly for lung adenocarcinoma in recent years, and our model was obviously associated with immunotherapeutic efficacy (Topalian et al., 2016; Hosseinkhani et al., 2020). We found high expression of immunoinhibitors and higher TMB scores in high-risk group, suggesting that immunotherapies may improve outcomes for patients with high risk scores. In addition, we performed a genetic mutation analysis of lung adenocarcinoma patients, which showed that patients with high risk scores had a higher mutation probability, revealing that lung adenocarcinoma patients with high risk scores were more likely to further progress. Moreover, it is noteworthy that we also predicted the efficacy of chemotherapies in our model, showing that IC_50_ values in high-risk group were significantly lower than in low-risk group in lung adenocarcinoma patients, suggesting that patients in the high-risk group were more sensitive to Camptothecin, Cisplatin, Rapamycin, Gemcitabine, Docetaxel, and Mitomycin C (Pirker, 2020). This indicated that our signature could be used for personalized treatment of LUAD patients.

Notably, we constructed a more accurate nomogram after integrating risk scores, age, gender, and pathological stages. In nomogram, the risk score was classified as an independent prognostic factor, which can be used as a complement to clinical factors. The risk score effectively took into account the missing parts of the pathological stage and improved the overall prediction effect of the signature. In general, a better understanding of cancer cell cycle control will help guide our treatment of patients. With a wide range of inhibitors of cell cycle checkpoints already in clinical studies, targeting cell cycle checkpoints is expected to be an important approach to cancer treatment (Ghelli Luserna di Rora’ et al., 2017; Gupta et al., 2022; Shcherba et al., 2014). However, there were still some shortcomings in our study which should be notified in generalizing the findings. First, our study was based on a bioinformatics approach which needs to be further validated in experiments. In addition, clinical applications of cell cycle checkpoints-related risk score and the constructed nomogram need to be validated in a clinical setting.

## Conclusion

In the current study, we used consensus clustering to identify two molecular subtypes based on cell cycle checkpoints-related genes in lung adenocarcinoma. Functional and immune analyses revealed that dysregulation of cell cycle checkpoints would hamper the immune system, affect cell cycle, and ultimately lead to poor prognosis in lung adenocarcinoma patients. In addition, we constructed a cell cycle checkpoints-related prognostic signature. This signature may be used for predicting prognosis and therapeutic response. To sum up, our study highlighted two cell cycle checkpoints related subtypes in LUAD and constructed a prognostic signature with four CCCRGs that can serve as a clinically useful indicator. Our work could contribute to risk stratification in lung adenocarcinoma patients, offer ideas for new targeted drugs, and provide theoretical support for personalized medicine.

## Data Availability

The original contributions presented in the study are included in the article/Supplementary Material, further inquiries can be directed to the corresponding authors.
