# Supplementary figures and images for: Immune Landscape and Classification in Lung Adenocarcinoma Based on a Novel Cell Cycle Checkpoints Related Signature for Predicting Prognosis and Therapeutic Response
Source: Front Genet. 2022 May 11;13:908104. doi: 10.3389/fgene.2022.908104 (PMC9130860; doi:10.3389/fgene.2022.908104)

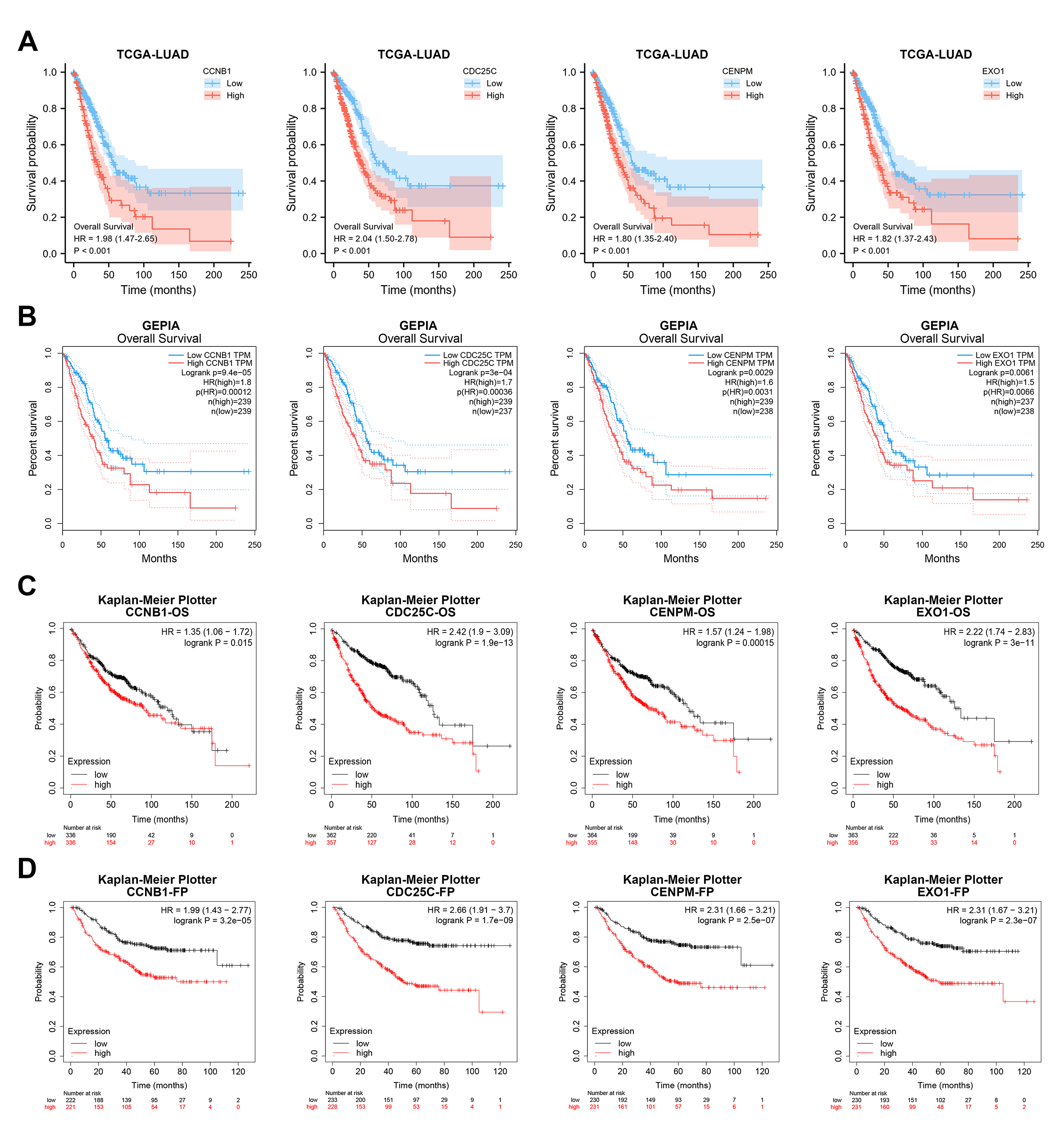

Supplement: Supplementary file 3 [file Image6.TIF]

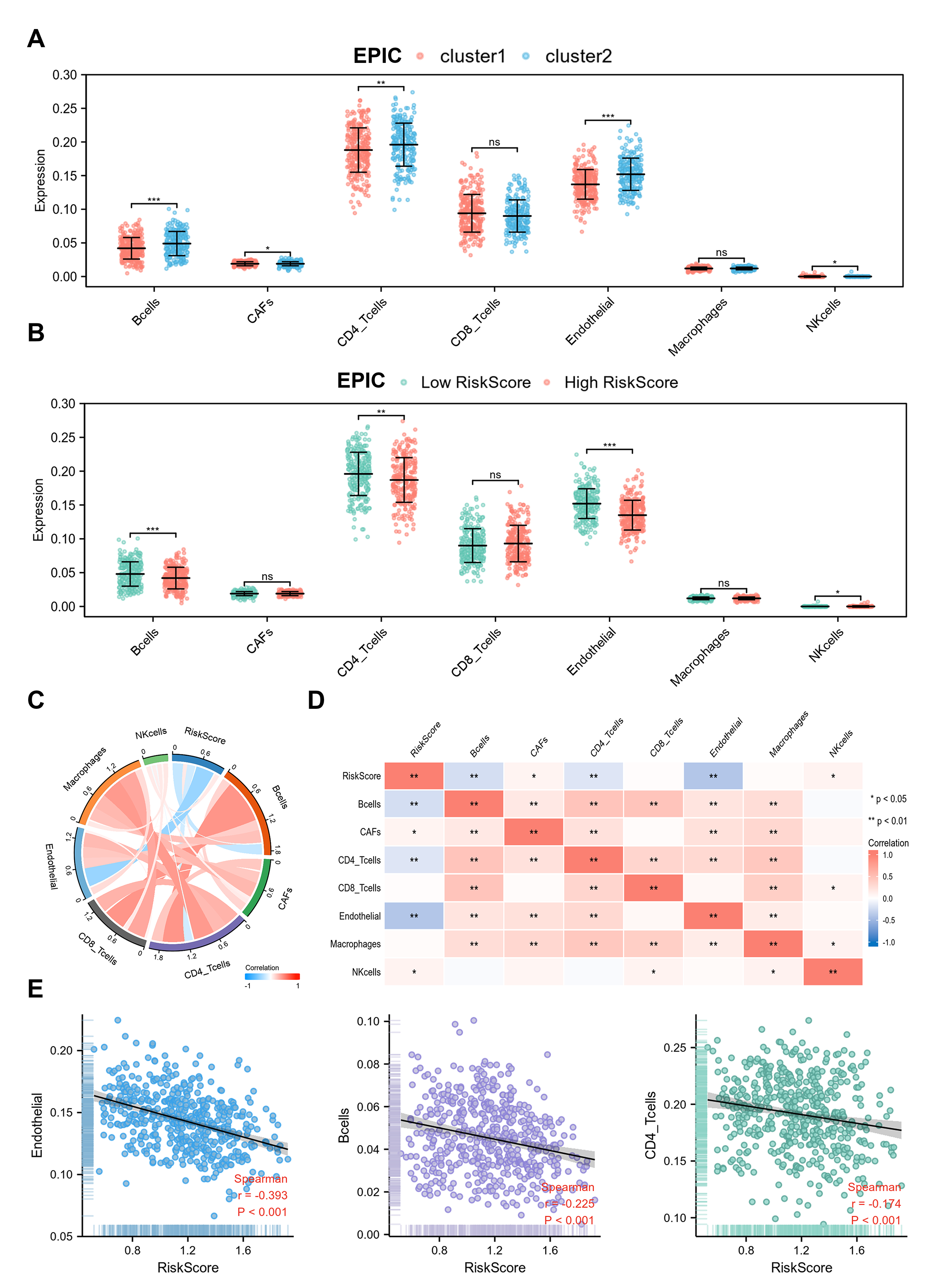

Supplement: Supplementary file 5 [file Image3.TIF]

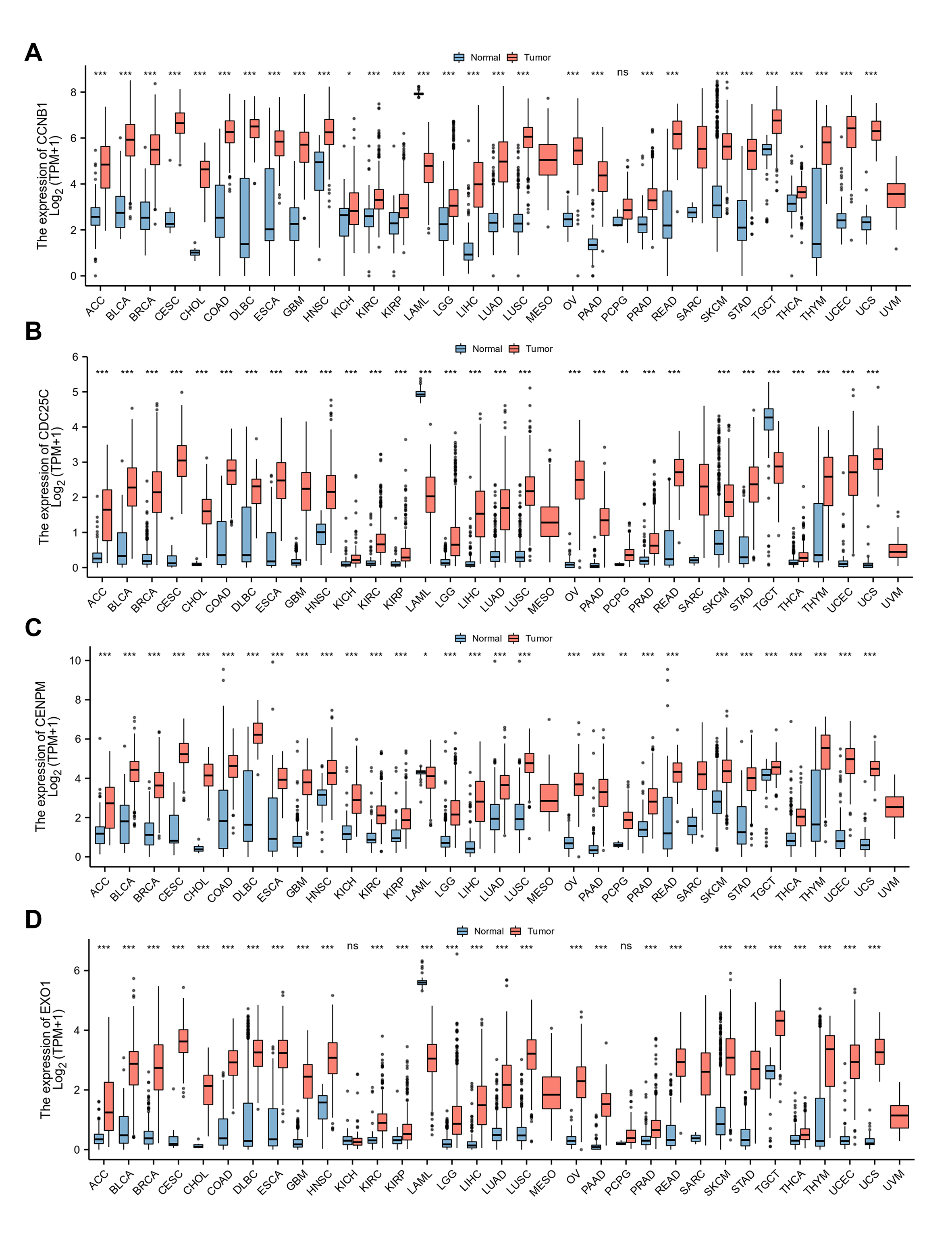

Supplement: Supplementary file 6 [file Image4.TIF]

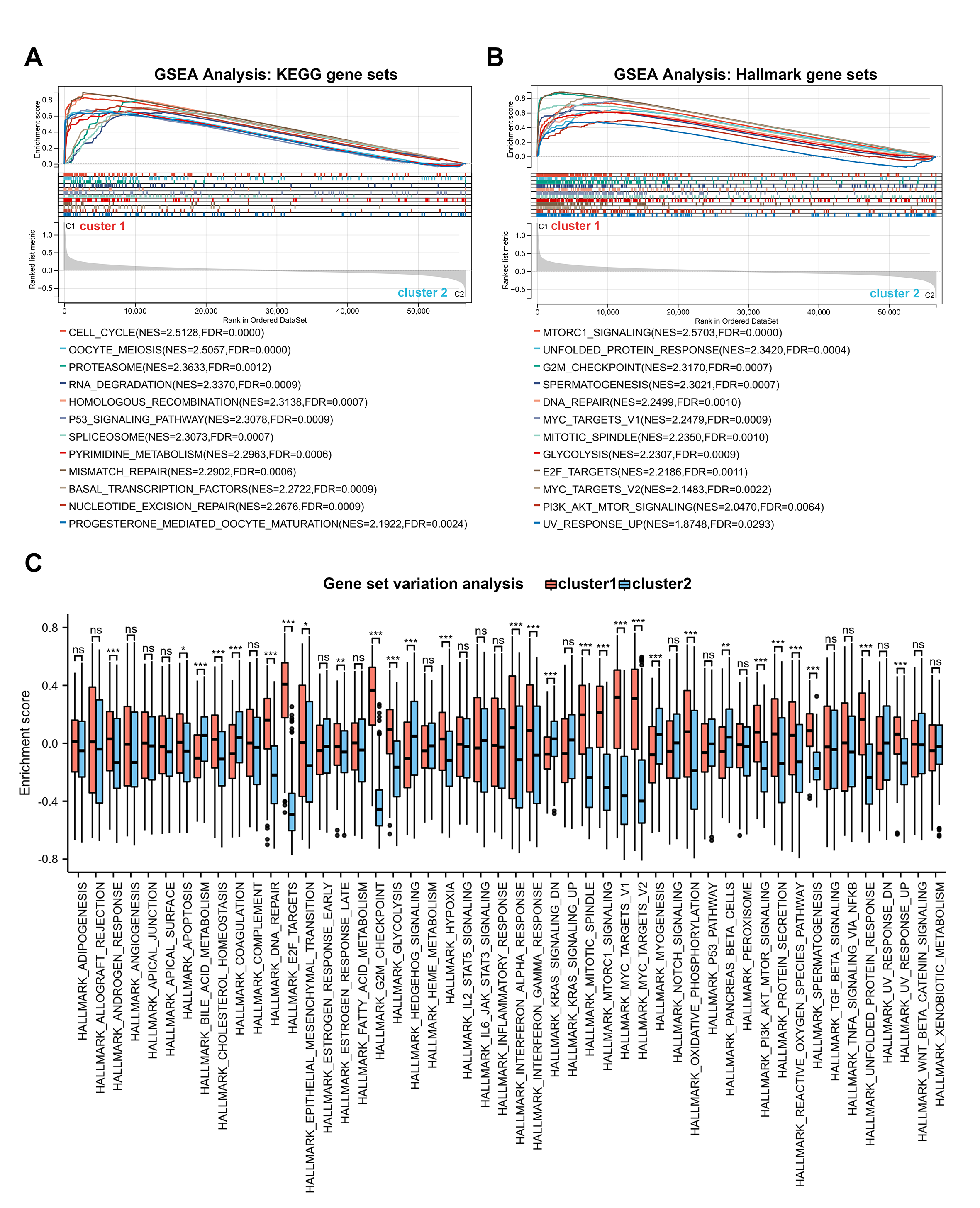

Supplement: Supplementary file 7 [file Image2.TIF]

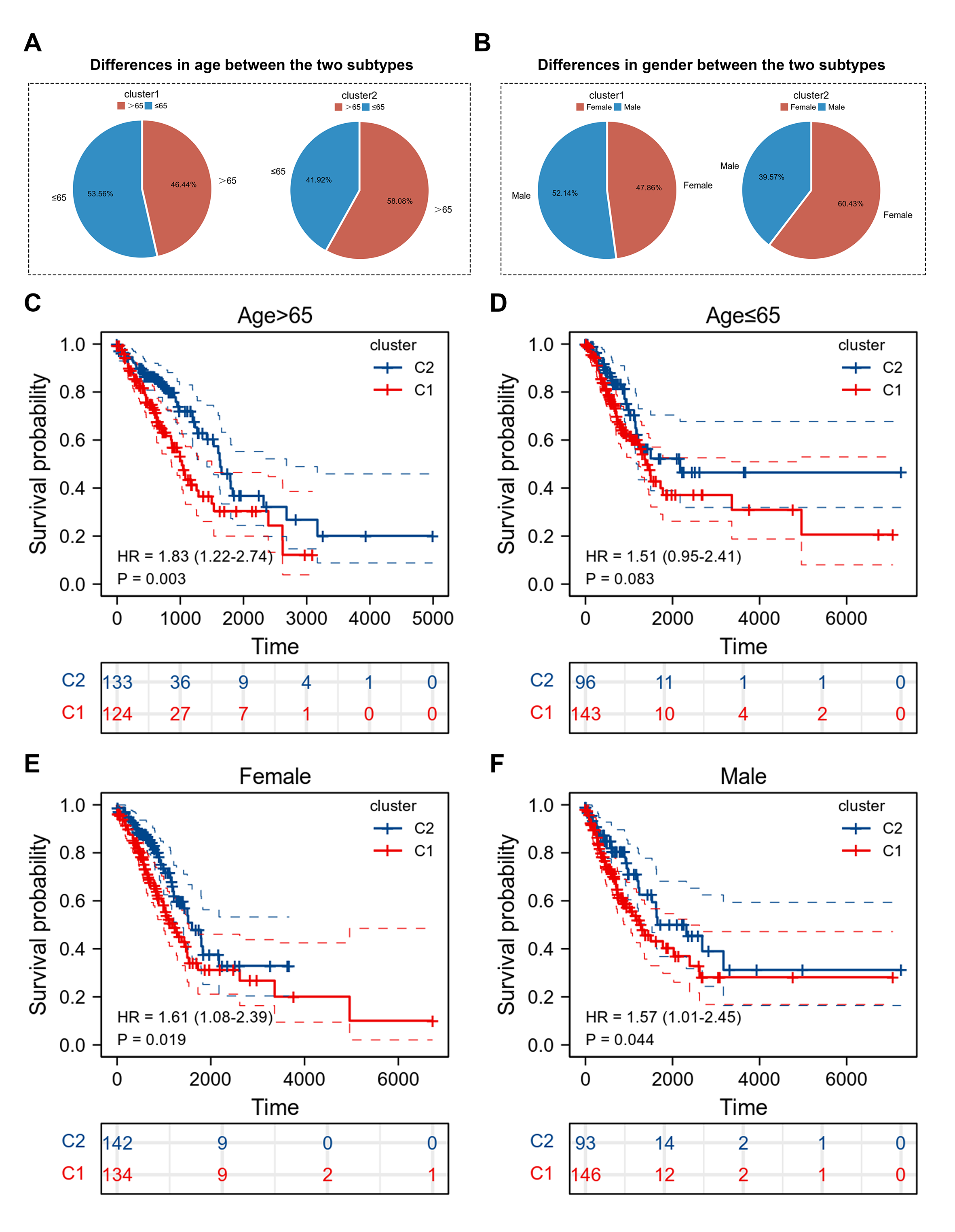

Supplement: Supplementary file 8 [file Image1.TIF]

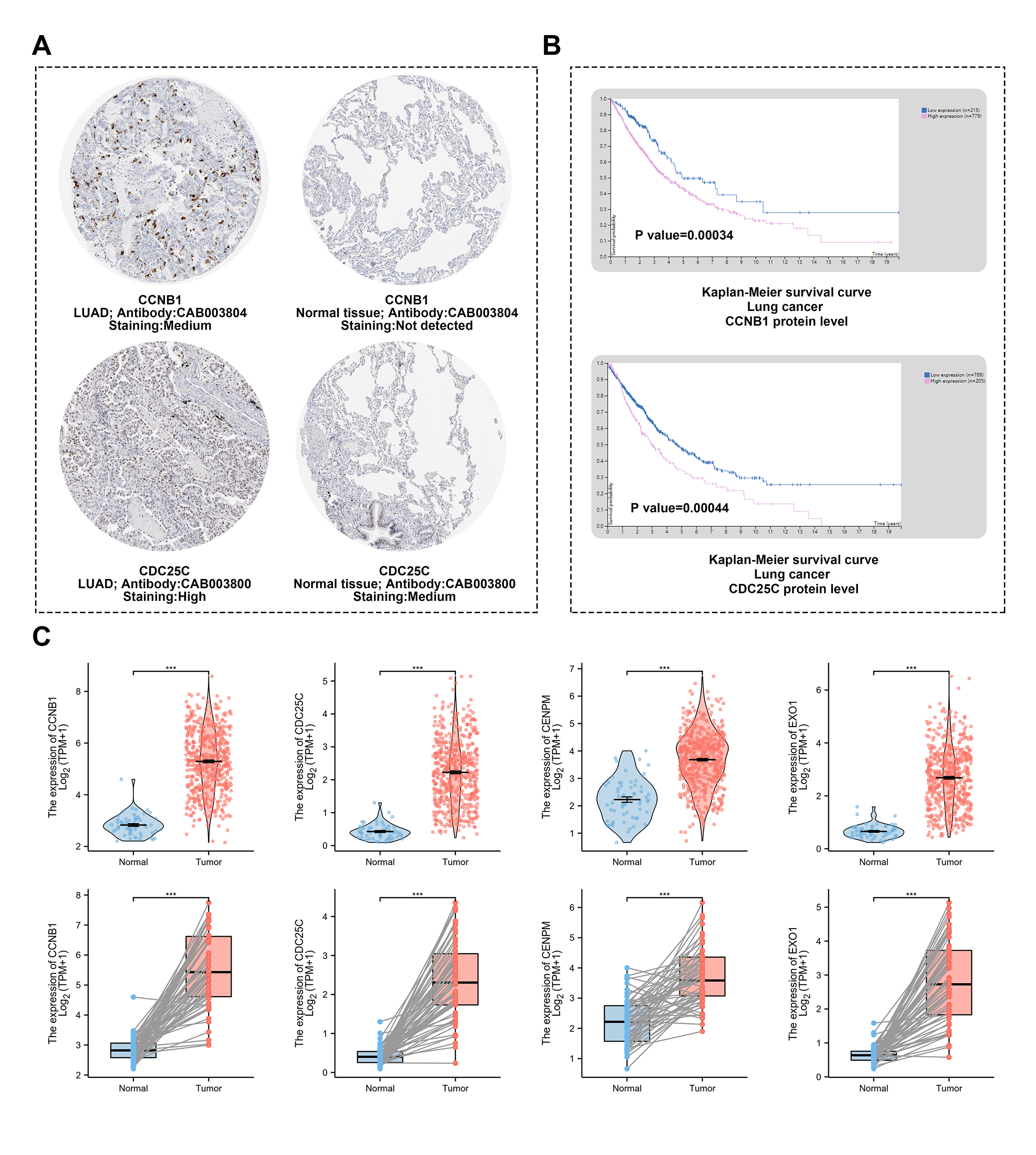

Supplement: Supplementary file 12 [file Image5.TIF]
