# Supplementary material for: Immune Landscape and Classification in Lung Adenocarcinoma Based on a Novel Cell Cycle Checkpoints Related Signature for Predicting Prognosis and Therapeutic Response
Source: Front Genet. 2022 May 11;13:908104. doi: 10.3389/fgene.2022.908104 (PMC9130860; doi:10.3389/fgene.2022.908104)
Supplement: Supplementary file 4 [file Table1.DOCX]

Supplementary Material

# Supplementary Figures and Tables

## Supplementary Figures

**Supplementary Figure 1.** Differences in age and gender between the two subtypes. (**A**) Differences in the proportions of the two subtypes in patients older than 65 years and those younger than or equal to 65 years. (**B**) Differences in the proportions of the two subtypes in gender. (**C-D**) Prognostic differences between two subtypes in age and gender.

**Supplementary Figure 2.** GSEA and GSVA enrichment analysis. (**A**) GSEA enrichment analysis of KEGG gene sets. (**B**) GSEA enrichment analysis of Hallmark gene sets. (**C**) GSVA enrichment analysis of 50 Hallmark gene sets.

**Supplementary Figure 3.** Immune infiltration levels calculated using EPIC algorithm. (**A**) Immune infiltration levels of seven immune cell types in two subtypes. (**B-E**) Correlation between immune infiltration levels and risk score in seven immune cell types.

**Supplementary Figure 4.** Pan-cancer analysis of four potential biomarkers. (**A-D**) The mRNA expression levels of CCNB1, CDC25C, CENPM, and EXO1 in the TCGA-GTEx database.

**Supplementary Figure 5.** Protein and mRNA expression levels of these biomarkers in lung adenocarcinoma. (**A-B**) Protein expression levels of CCNB1, CDC25C in lung adenocarcinoma and their association with prognosis. (**C**) The mRNA expression levels of CCNB1, CDC25C, CENPM, and EXO1 in the TCGA-LUAD database.

**Supplementary Figure 6.** Prognostic significance of CCNB1, CDC25C, CENPM, EXO1 in TCGA-LUAD (**A**), GEPIA (**B**), Kaplan-Meier Plotter (**C-D**) databases.

## Supplementary Tables

**Supplementary Table 1.** KEGG analysis of differentially expressed genes.

**Supplementary Table 2.** GO analysis of differentially expressed genes.

**Supplementary Table 3.** GSEA enrichment analysis of KEGG gene sets.

**Supplementary Table 4.** GSEA enrichment analysis of Hallmark gene sets.

**Supplementary Table 5.** Fifty Hallmark gene sets.
