# Supplementary material for: Immune Landscape and Classification in Lung Adenocarcinoma Based on a Novel Cell Cycle Checkpoints Related Signature for Predicting Prognosis and Therapeutic Response
Source: Front Genet. 2022 May 11;13:908104. doi: 10.3389/fgene.2022.908104 (PMC9130860; doi:10.3389/fgene.2022.908104)
Supplement: Supplementary file 10 [file Table5.DOCX]

| Term | ES | NES | pvalue | FDR | FWER |
| --- | --- | --- | --- | --- | --- |
| UNFOLDED_PROTEIN_RESPONSE | 0.6481 | 2.342 | 0.0004 | 0.001 | 0 |
| G2M_CHECKPOINT | 0.8658 | 2.317 | 0.0007 | 0.002 | 0 |
| SPERMATOGENESIS | 0.6402 | 2.3021 | 0.0007 | 0.003 | 0 |
| DNA_REPAIR | 0.6253 | 2.2499 | 0.001 | 0.004 | 0 |
| MYC_TARGETS_V1 | 0.76 | 2.2479 | 0.0009 | 0.004 | 0 |
| MITOTIC_SPINDLE | 0.7098 | 2.235 | 0.001 | 0.004 | 0 |
| GLYCOLYSIS | 0.6096 | 2.2307 | 0.002 | 0.0009 | 0.004 |
| E2F_TARGETS | 0.8856 | 2.2186 | 0.0011 | 0.006 | 0 |
| MYC_TARGETS_V2 | 0.8148 | 2.1483 | 0.0022 | 0.015 | 0 |
| PI3K_AKT_MTOR_SIGNALING | 0.4958 | 2.047 | 0.004 | 0.0064 | 0.046 |
| UV_RESPONSE_UP | 0.4757 | 1.8748 | 0.01 | 0.0293 | 0.151 |

**Supplementary Table 4.** GSEA enrichment analysis of Hallmark gene sets (FDR<0.05)
