# Supplementary material for: Immune Landscape and Classification in Lung Adenocarcinoma Based on a Novel Cell Cycle Checkpoints Related Signature for Predicting Prognosis and Therapeutic Response
Source: Front Genet. 2022 May 11;13:908104. doi: 10.3389/fgene.2022.908104 (PMC9130860; doi:10.3389/fgene.2022.908104)
Supplement: Supplementary file 11 [file Table4.DOCX]

**Supplementary Table 3.** GSEA enrichment analysis of KEGG gene sets (FDR<0.05)

| **Term** | **ES** | **NES** | **pvalue** | **FDR** | **FWER** |
| --- | --- | --- | --- | --- | --- |
| **CELL_CYCLE** | **0.8236** | **2.5128** | **0** | **0** | **0** |
| **OOCYTE_MEIOSIS** | **0.6664** | **2.5057** | **0** | **0** | **0** |
| **PROTEASOME** | **0.7897** | **2.3633** | **0.0012** | **0.003** | **0** |
| **RNA_DEGRADATION** | **0.6433** | **2.337** | **0.0021** | **0.0009** | **0.003** |
| **HOMOLOGOUS_RECOMBINATION** | **0.8704** | **2.3138** | **0.0007** | **0.003** | **0** |
| **P53_SIGNALING_PATHWAY** | **0.6768** | **2.3078** | **0.0009** | **0.005** | **0** |
| **SPLICEOSOME** | **0.6883** | **2.3073** | **0.0021** | **0.0007** | **0.005** |
| **PYRIMIDINE_METABOLISM** | **0.6507** | **2.2963** | **0.0006** | **0.005** | **0** |
| **MISMATCH_REPAIR** | **0.8903** | **2.2902** | **0.0006** | **0.005** | **0** |
| **BASAL_TRANSCRIPTION_FACTORS** | **0.7032** | **2.2722** | **0.0009** | **0.009** | **0** |
| **NUCLEOTIDE_EXCISION_REPAIR** | **0.7243** | **2.2676** | **0.0009** | **0.009** | **0** |
| **PROGESTERONE_MEDIATED_OOCYTE_MATURATION** | **0.639** | **2.1922** | **0.0024** | **0.025** | **0** |
| **DNA_REPLICATION** | **0.9017** | **2.1781** | **0.0026** | **0.027** | **0** |
| **UBIQUITIN_MEDIATED_PROTEOLYSIS** | **0.5457** | **2.108** | **0.0063** | **0.0077** | **0.061** |
| **CYSTEINE_AND_METHIONINE_METABOLISM** | **0.5827** | **2.0805** | **0.002** | **0.0096** | **0.073** |
| **RNA_POLYMERASE** | **0.6679** | **2.043** | **0.004** | **0.0125** | **0.093** |
| **AMINOACYL_TRNA_BIOSYNTHESIS** | **0.6672** | **2.039** | **0.0062** | **0.0125** | **0.097** |
| **PENTOSE_PHOSPHATE_PATHWAY** | **0.6239** | **1.9556** | **0.0117** | **0.0242** | **0.148** |
| **NON_HOMOLOGOUS_END_JOINING** | **0.7238** | **1.9089** | **0.0041** | **0.034** | **0.195** |
| **PURINE_METABOLISM** | **0.4714** | **1.9064** | **0.0041** | **0.033** | **0.197** |
| **GLYOXYLATE_AND_DICARBOXYLATE_METABOLISM** | **0.664** | **1.8805** | **0.0103** | **0.0392** | **0.228** |
| **PATHOGENIC_ESCHERICHIA_COLI_INFECTION** | **0.5219** | **1.8692** | **0.018** | **0.0411** | **0.24** |
| **PANCREATIC_CANCER** | **0.5441** | **1.8636** | **0.032** | **0.0416** | **0.249** |
| **ARACHIDONIC_ACID_METABOLISM** | **-0.6607** | **-2.1116** | **0.0522** | **0.046** | **0** |
